# Microbial succession and the functional potential during the fermentation of Chinese soy sauce brine

**DOI:** 10.3389/fmicb.2014.00556

**Published:** 2014-10-31

**Authors:** Joanita Sulaiman, Han Ming Gan, Wai-Fong Yin, Kok-Gan Chan

**Affiliations:** ^1^Division of Genetics and Molecular Biology, Institute of Biological Sciences, Faculty of Science, University of MalayaKuala Lumpur, Malaysia; ^2^School of Science, Monash University MalaysiaPetaling Jaya, Malaysia

**Keywords:** traditional Chinese soy sauce, fermentation, metagenomic, next generation sequencing, whole genome shotgun, food microbiology

## Abstract

The quality of traditional Chinese soy sauce is determined by microbial communities and their inter-related metabolic roles in the fermentation tank. In this study, traditional Chinese soy sauce brine samples were obtained periodically to monitor the transitions of the microbial population and functional properties during the 6 months of fermentation process. Whole genome shotgun method revealed that the fermentation brine was dominated by the bacterial genus *Weissella* and later dominated by the fungal genus *Candida*. Metabolic reconstruction of the metagenome sequences demonstrated a characteristic profile of heterotrophic fermentation of proteins and carbohydrates. This was supported by the detection of ethanol with stable decrease of pH values. To the best of our knowledge, this is the first study that explores the temporal changes in microbial successions over a period of 6 months, through metagenome shotgun sequencing in traditional Chinese soy sauce fermentation and the biological processes therein.

## INTRODUCTION

Soy sauce is an important condiment in Asian food and the quality of soy sauce is dependent on the microbial population during its fermentation process (Yong and Wood, 1974; O’toole, 1997; Hutkins, 2006). Unlike other soy sauce production, the fermentation process of traditional Chinese soy sauce, both its solid and liquid states, do not involve defined inoculum. Traditional Chinese soy sauce fermentation manufacturing relies solely on natural microbial selection to produce the end product. The microbial assemblage in these traditional processes is more complex than that of controlled industrialized food (Ercolini, 2013). Additionally, the usage of starter cultures under mass production fermenters fail to duplicate the whole microbial community of traditional fermentation. This leads to the production of less fragrant products (Zhao et al., 2009). Therefore, determining the whole microbial populations involved in the traditional fermentation processes will assist in the development of define Chinese soy sauce culture, which would allow future improvement in production output whilst still maintaining the required qualities.

Previous studies on microbial communities in soy based fermentation processes using PCR-denaturing gradient gel electrophoresis (PCR-DGGE) reported the presence of *Weissella cibaria*, *Lactobacillus fermentum, Staphylococcus gallinarium, Staphylococcus kloosii,* and *Staphylococcus arlette* (Kim et al., 2010; Tanaka et al., 2012). Similar studies using pyrosequencing noted that Korean fermented soybean bricks (*Meju*) were dominated by the lactic acid bacteria (LAB) and *Bacillus* species (Kim et al., 2011). Although culture independent protocols, such as the PCR-DGGE and 16S rRNA genes metagenome sequencing provided insights into the general fermentative microbial population and environment, they introduce bias in determining microbial abundance (Polz and Cavanaugh, 1998; Nikolausz et al., 2005; Hong et al., 2009). In contrast, whole genome shotgun (WGS) analysis provides useful data pertaining to not only the taxonomic perspective, but also the metabolic and functional diversity of the microbial population during fermentative stages (Eisen, 2007; Simon and Daniel, 2009). WGS therefore, provides a better view of the fermentation process.

The quality of soy sauce is determined by its chemical properties (Yong and Wood, 1974; Gao et al., 2011). The chemical changes within the fermentation liquor selectively inhibit the growth of certain organisms, while allowing others to proliferate (Noda et al., 1980; Hutkins, 2006; Kim et al., 2011). By analyzing the composition of the microbial community and chemical components involved, interrelationship, and functional profiles can be established.

Prior to this, no systematic study on the mixed microbial community of traditional Chinese soy sauce using shotgun metagenome approach has been done. By utilizing the WGS approach coupled with next-generation sequencing (NGS), this study aims to determine the succession of bacterial and fungal communities and its potential metabolic capabilities during Chinese soy sauce fermentation.

## MATERIALS AND METHODS

### SAMPLE COLLECTION

Chinese soy sauce fermentative samples were taken from a fermentation tank of traditional Chinese soy sauce factory in Malaysia. The sample does not involve commercial starter culture. The soy sauce fermentation brine (3.0 L) was collected from the same tank at day zero, 1, 2, 3, 4, 5, and 6 months of fermentation. Samples were collected in sterile bottles and stored at -20∘C prior to DNA extraction and physicochemical analysis.

### PHYSICOCHEMICAL ANALYSIS OF SOY SAUCE BRINE

Total nitrogen and salt concentration was tested using the Kjeldahl method and direct titration method based on Mohr method, respectively. Ethanol concentration was determined by gas chromatography (GC) and total acidity was measured based on titration method. The reducing sugar levels were determined using the Lane-Eynon titration method. All the above analysis except the pH determination, were outsourced to accredited ISO 17025 certified laboratories.

### METAGENOMIC DNA EXTRACTION AND NGS

The soy sauce brine was centrifuged at 10,000 *g* for 45 min at 4∘C. The pellets were washed three times with phosphate-buffered saline (PBS; pH 7.0) and filtered through four layers of sterile gauze. The filtrate were sequentially filtered through nitrose cellulose membranes (1.2 and 0.2 μm pore sizes, respectively) using a vacuum pump. The DNA was harvested from the membranes and was extracted using Metagenome DNA Isolation Kit for Water (Epicentre, USA) with minor modifications. The extracted DNA was further purified using the Agencourt AMPure XP (Beckman Coulter, USA) according to the manufacturer’s protocol. DNA quality was checked on NanoDrop 2000c instrument (Thermo Scientific, USA) and quantified on *Qubit* 2.0 Fluorometer (Invitrogen, USA). Sequence libraries were generated with the total genomic DNA using Nextera DNA Sample Preparation Kits (Illumina, USA) according to the manufacturer’s guidelines. The libraries were quantified using *Qubit* 2.0 Fluorometer. The quality and the size distribution of the libraries were determined by using High Sensitivity DNA chips and DNA Reagents on BioAnalyzer 2100 (Agilent, USA). Sequencing was performed in the MiSeq (Illumina, USA) system for a 151 cycle paired-end run.

### SEQUENCE ANALYSIS OF EXTRACTED SOY SAUCE METAGENOMIC DNA

The raw sequences were trimmed to Qscore of 20 and then assembled with CLC Genomic Workbench 5.1.1. Sequences less than 50 bp were discarded. Minimum contig length was set at 400 bp. Assembled contigs with an average coverage of more than 10 times were searched for the presence of eukaryotic components by BLASTN with GenBank nucleotide (nt) database with e-value of <10^-6^. The contigs were then segregated into prokaryote and eukaryotic origins. The quality trimmed unassembled reads were subjected to USEARCH v6.0 (Edgar, 2010) with the SSURef_111_NR alignment from SILVA (Pruesse et al., 2007) for both the 16S rRNA and 18S rRNA genes (e-value of <10^-20^). The USEARCH outputs from each sample were normalized internally in MEGAN version 4.70.4 by checking the “use normalized count” option prior to combining the data for biodiversity and abundance assessment (Huson et al., 2011). Taxonomic classification was based on 97% identity threshold, at genus level. Microbial relative abundance was calculated based on the proportion of assigned reads within each fermentation stage. In order to compare the overall microbial community in each of the soy sauce brine fermentation stages and the sampling completeness, the sequences from all of the samples were clustered into OTUs based on 97% sequence similarity with uclust and Good’s coverage, respectively, using an open source software, Quantitative Insights into Microbial Ecology (QIIME; Caporaso et al., 2010).

Genes were predicted on the prokaryotic contigs using Prodigal (Prokaryotic Dynamic Programming Genefinding Algorithm; Hyatt et al., 2010). Separately, the gene predictions of the eukaryotic contigs were done using web server gene prediction software, AUGUSTUS (Stanke and Morgenstern, 2005). The predicted genes were compared to reference protein sequences in NCBI non-redundant (nr) database using protein database search tool RAPSearch V2_60 (Ye et al., 2011). Functional analysis was done based on the Kyoto encyclopedia of genes and genomes (KEGG) classification scheme using MEGAN version 4.70.4. Metabolic pathways present were filtered using minimal set of pathways (MinPath) parsimony analysis (Ye and Doak, 2009). Statistical analysis was done based on the number of coding sequences (CDS) categorized in the functional categories. SPSS version 21 (SPSS IBM, USA) was used to perform one way ANOVA analysis to determine the mean difference of CDS between all functional categories. STAMP analysis was further employed to perform statistical analysis of gene enrichment in each functional categories using the default setting for two-samples comparison, e.g., two-sided *G*-test (w/Yates’) + Fisher’s exact test (Parks et al., 2014).

In order to establish a traditional Chinese soy sauce core metagenome, unassembled quality trimmed reads from all seven samples were combined and assembled via *de novo* assembly. The abundance of a specific gene of interest was calculated by mapping reads from each sample to the draft metagenome with 90% identity cutoff and at least 80% aligned length. The functional potential was then visualized using the KEGG classification scheme. The genes encoding for the enzymes class of interest were inspected and the sequence descriptions were verified using Blast2GO (Conesa et al., 2005). The gene abundance was estimated by dividing the accumulated abundance value (total reads/gene length) of genes in each selected enzyme commission (EC) with the total number of quality trimmed reads. The value was subsequently multiplied with one million to obtain reads per bases per million reads

### NUCLEOTIDE SEQUENCES ACCESSION NUMBER

The soy sauce brine metagenomic raw reads were deposited in the NCBI Short Read Archive with accession number SRA064709 under study accession SRP017928.

## RESULTS

### PHYSICOCHEMICAL PROPERTIES OF THE SOY SAUCE BRINE

**FIGURE 1:**
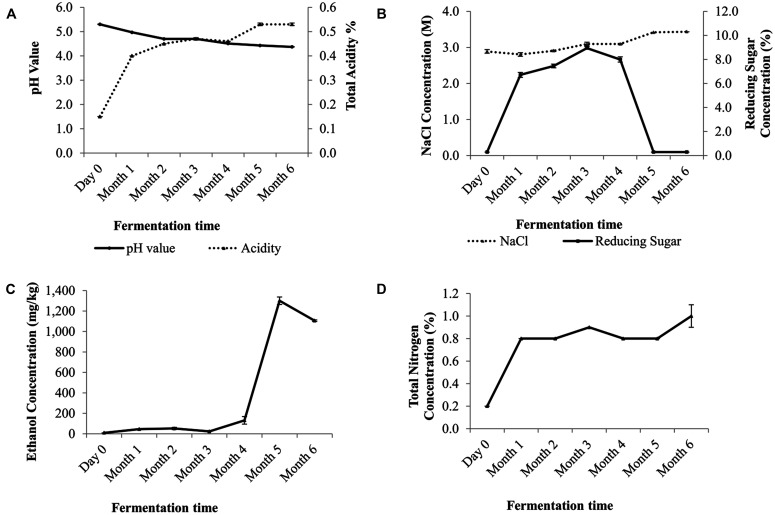
**Physicochemical changes in the traditional Chinese soy sauce fermentation brine (A) pH and acidity mean values at various fermentation stages.** Data are expressed as the means of ± SEM value of triplicates (pH value) and duplicates (acidity), **(B)** NaCl and reducing sugar content, **(C)** ethanol concentration of traditional Chinese soy sauce and **(D)** changes in percentages of total nitrogen content. Data are presented as means ± SEM values of duplicates.

The pH value showed a gradual decrease over time. At day zero, the initial pH value of pH 5.3 at day zero decreased to pH 4.3 on the sixth month. The total acidity content increased steadily from 0.15% (w/v) at day zero to 0.53% (w/v) at month six (**Figure 1A**). The reducing sugar level peaked at the third month and decreased to less than 0.3% (w/v) by the end of the fermentation (**Figure 1B**). The soy sauce mash salt concentration was at 2.89 M at day zero and ended at 3.44 M (**Figure 1B**). Ethanol concentration was not detected at the beginning but began to increase from the fourth month onward and achieved its maximal level of 0.13% (w/w) by the fifth month. By the end of the fermentation, the ethanol concentration was at 0.1% (w/w; **Figure 1C**). The total nitrogen level began to increase after a month into the fermentation process and was at 1% by the end of the sixth month (**Figure 1D**).

### NGS USING ILLUMINA MiSeq

A total of 11.36 Gb of raw reads were generated from seven soy sauce fermentation brine samples. The quality reads after trimming was 9.10 Gb of sequences. An average of 1.30 Gb (ranging between 1.20 to 1.96 Gb) of quality sequences was obtained from each sample. The average length of sequence generated after quality trim was between 112.2 to 134.2 bp. **Table 1** summarizes the NGS data statistics from the soy sauce samples. The assembled sequences from all seven stages of fermentation generated a total of 63,245 contigs (86.02 Mb) of more than 400 bp in length, with N50 of 1,873 bp. More than 85% of the genes had at least 50% of their length covered by a single fermentation stage at 80% identity threshold. This indicated that the gene set from each fermentation stage includes more than half of the fermentation process genes.

**Table 1:** Summary of MiSeq Illumina sequences.

	Day 0	Month 1	Month 2	Month 3	Month 4	Month 5	Month 6
No. of sequences generated (bp)	1,774,294,619	1,201,774,458	1,806,125,496	1,424,367,836	1,956,458,463	1,500,286,688	1,694,203,762
No. of reads generated	15,674,350	10,434,346	11,961,096	10,526,436	13,725,540	10,749,596	11,777,702
No. of quality sequences generated (bp)	1,505,234,972	1,078,183,875	1,182,280,801	1,210,597,794	1,582,353,972	1,213,475,521	1,316,079,598
No. of quality reads	13,976,473	9,482,454	10,820,621	9,630,743	12,420,866	9,545,509	10,550,831
Avg. length of quality reads (bp)	112.2	116.4	113.5	130.5	134.2	133.4	132.2
% of reads mapping to 16S rRNA^1^	0.67	1.0	0.77	0.55	0.25	0.38	0.23
% of reads mapping to 18S rRNA^1^	0.01	0.01	0.10	0.37	0.44	0.52	0.57
No. of contig (>400 bp)	24,012	17,560	26,767	23,299	23,091	20,013	16,247
Total contig lengths (bp)	30,520,799	21,335,799	41,751,918	42,649,821	41,669,126	36,317,955	28,098,581

*^1^Percentages calculated based on the total number of quality reads after removing sequences <50 bp.*

### TAXONOMIC ASSIGNMENTS OF SOY SAUCE MICROBIOME

At 97% confidence threshold, an average of 0.55% of the reads matched the 16S rRNA genes, and 0.29% of the reads matched the 18S rRNA genes (**Table 1**) while 0.08% of the reads were unassigned. The analysis indicated the number of 16S rRNA genes decreased whereas 18S rRNA increased as the fermentation progressed. No Archaeal 16S rRNA genes were found.

**FIGURE 2:**
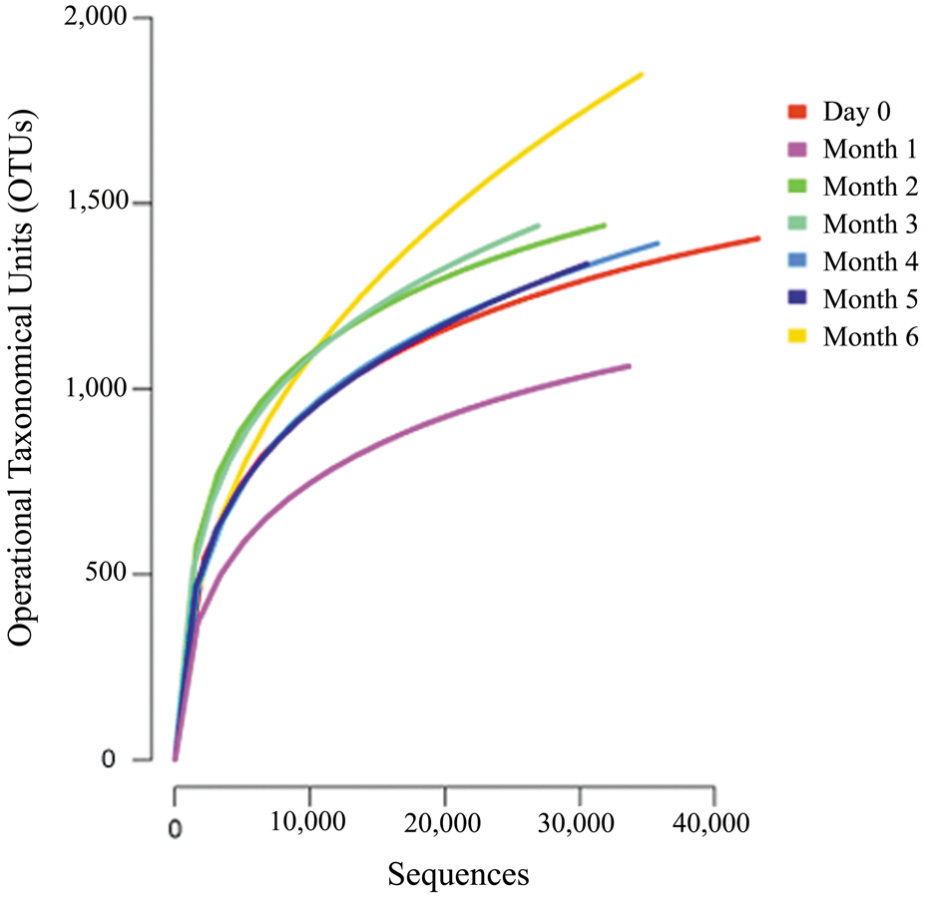
**Rarefaction curves of the microbial diversity in traditional Chinese soy sauce fermentation brine.** Rarefaction curves were constructed with 97% sequence similarity at genus level.

The average number of operational taxonomical units (OTUs) for each sample was 1360, which ranged between 1040 and 1800 OTUs. A rarefaction analysis was used to determine the sequence coverage of the current study. The result showed similar curves pattern from each of the stages without reaching saturation (**Figure 2**). This suggests that a portion of OTUs still existed and that more sequencing attempts are required to detect all the available phylotypes. However, based on Good’s coverage at 97% threshold, estimated the sampling completeness at an average of 98.9% (ranging in between 98 to 99%). This suggests that the majority of the microbial phylotypes in the traditional Chinese soy sauce sample were identified.

**FIGURE 3:**
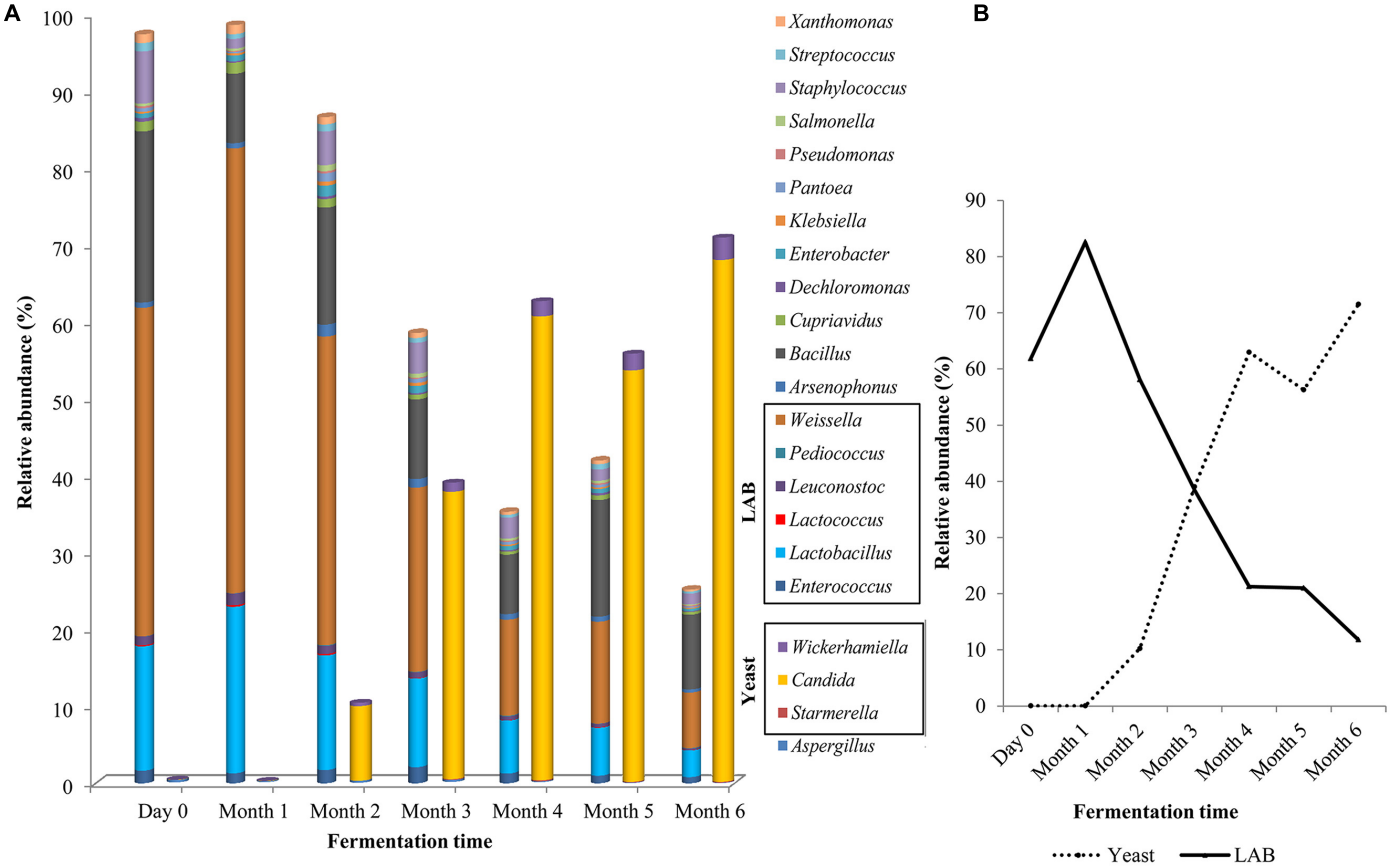
**Microbial diversity of traditional Chinese soy sauce fermentation brine **(A)** Relative abundance of 22 frequent microbial genus and **(B)** Relative abundance of yeast and LAB in soy sauce mash throughout the fermentation process**.

A microbial succession was observed over the 6-month fermentation process (**Figure 3A**). At day zero, *Weissella* (42%) dominated the fermentation brine, followed by *Bacillus* and *Lactobacillus* with 22 and 16%, respectively (**Figure 3A**). These three genera remained the most abundant population in the soy sauce fermentation brine until the third month and declined by the fourth month, to a total of 19% by the end of sixth month. Throughout the fermentation process, LAB (*Weissella, Lactobacillus, Leuconostoc, Pediococcus, Enterococcus,* and *Lactobacillus*) contributed more than 43% of the population (**Figure 3A**).

The relative abundance of the yeast community began to increase in the second month of fermentation. *Candida* accounted for only 0.03% of the population at day zero but by the sixth month, the *Candida* population represented more than 68%. The change in the dominancy of yeast and LAB population are presented in **Figure 3B**. Overall, the biodiversity of bacterial population of the fermentation stages maintained throughout the process and increased in the final stage of fermentation. On the contrary, the diversity of fungal population displayed almost twofold-lower Shannon-Weaver index from 2.166 at day zero to 0.33 by the end of the fermentation process.

### MICROBIAL FUNCTIONS IN TRADITIONAL CHINESE SOY SAUCE BRINE FERMENTATION

**FIGURE 4:**
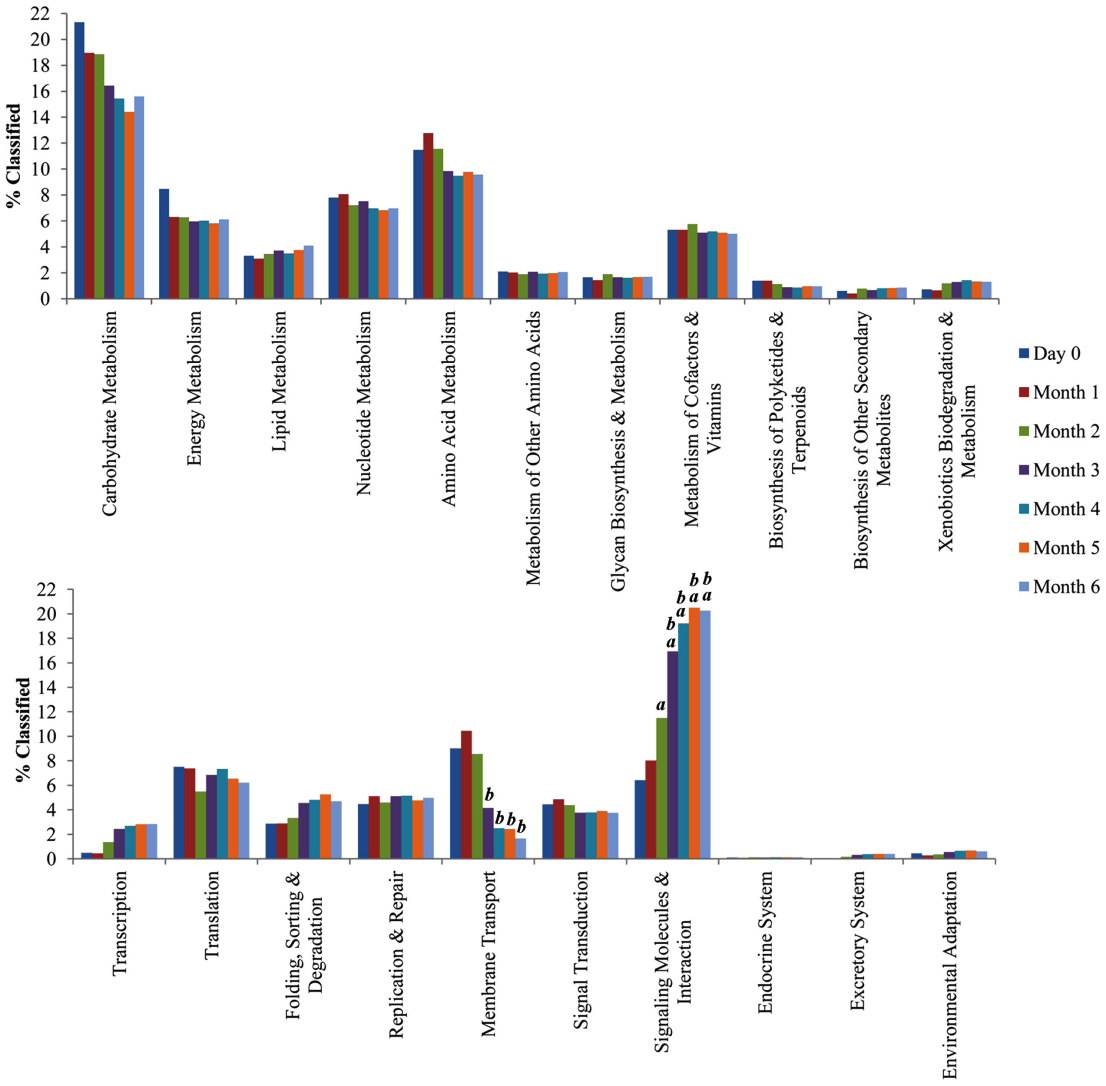
**Traditional Chinese soy sauce fermentation brine functional capabilities and diversities.** % Classified (Y-axis) represents the percentage of annotated predicted proteins from the metagenome assembly assigned to different Kyoto encyclopedia of genes and genomes (KEGG) second-level pathways. Letters “a” and “b” above bar indicates significant difference (*p*-value < 0.05) in comparison to Month 0 and Month 1 respectively.

A total of 176 predicted pathways were identified in this study, enabling better understanding toward the microbial functional capabilities. There was significant mean difference of CDS among the functional categories by *post hoc* test Bonferroni’s procedures. The metabolic reconstruction using KEGG showed an average of 3872 CDS (16.96%; *p* < 0.001) was classified under carbohydrate metabolism and was consistently found in all of the soy sauce metagenome libraries. Amino acid metabolism, nt metabolism, and energy metabolism showed an average of 2404 CDS (10.53%; *p* < 0.01), 1666 CDS (7.29%; *p* < 0.05), and 1441 CDS (6.31%; *p* < 0.01), respectively (**Figure 4**). Additional statistical analysis using STAMP also showed significant enrichment genes pertaining to membrane transport and signaling molecules and interaction in the later stage of the fermentation.

The overall reconstruction showed the presence of glycolysis, citrate cycle (TCA cycle), and pentose phosphate pathway among other carbohydrate metabolism pathways. The annotated functions are important metabolic pathways for all bacteria and protein complexes (oxidative phosphorylation), in addition to cellular metabolism (purine and pyrimidine metabolism). Upon closer inspection into the third level of the KEGG pathway, reputed fermentation specific functions were found, which included the production of flavoring and aromatic compounds (arginine, proline, alanine, aspartate, glutamate). Further examination into the orthologous gene families led to the findings of three unique functions to the pathway, namely EC 4.4.1.22 [*S*-(*hydroxymethyl*) glutathione synthase], EC 1.1.1.284 [*S*-(*hydroxymethyl*) glutathione dehydrogenase], and EC 3.1.2.12 (*S*-formylglutathione hydrolase). These enzymes are important in the detoxification of formaldehyde in bacteria (Goenrich et al., 2002).

**FIGURE 5:**
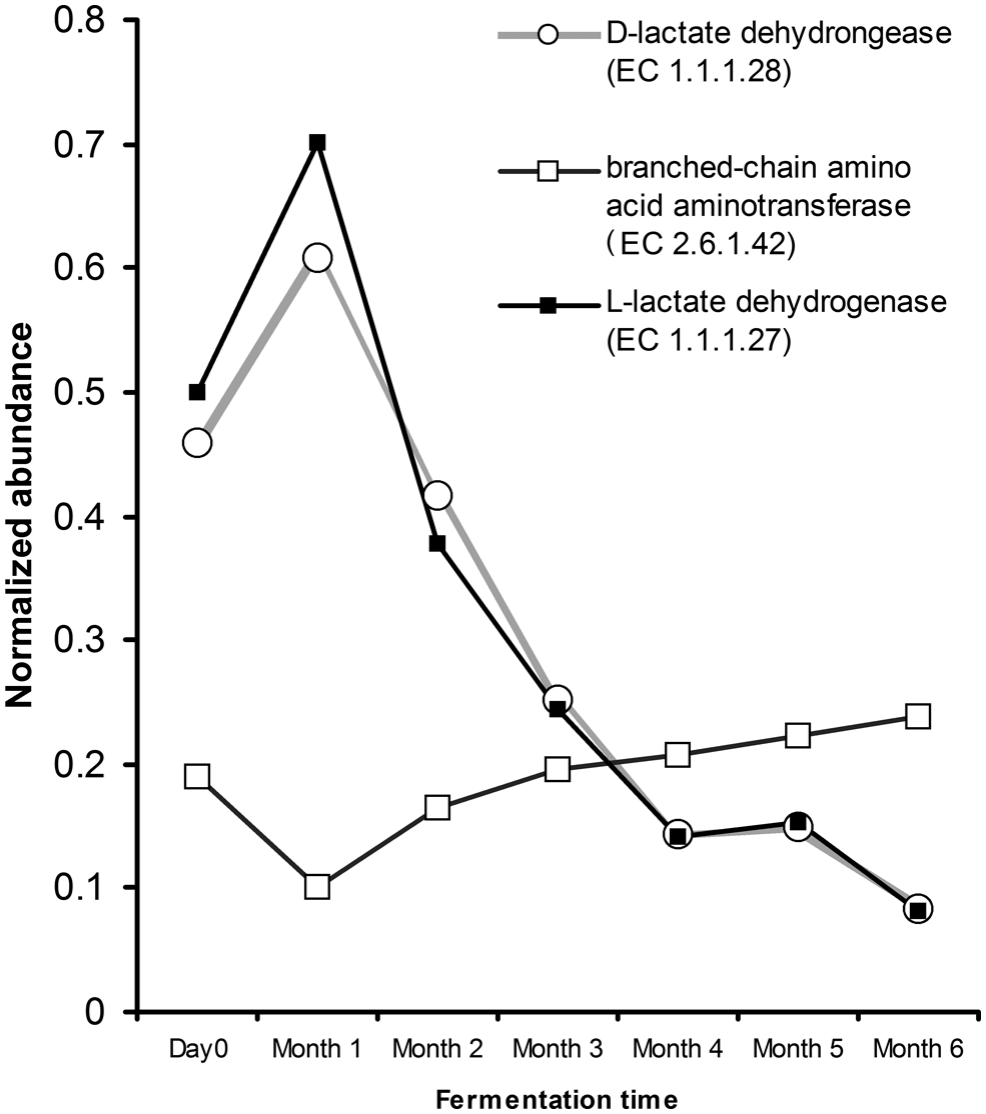
**Abundance estimation of genes assigned to lactate dehydrogenase (EC 1.1.1.27 and 1.1.1.28) and branched-chain amino acid transferase (EC 2.6.1.42) relevant to soy sauce fermentation.** Gene abundance was normalized to the number of reads and gene length.

Inspection into the metabolic potential of traditional Chinese soy sauce revealed the microbial capability for adaptation. The genes involved in oxidative phosphorylation revealed the presence of cytochrome *bd* complex. The presence of cytochrome *bd* complex enhances microbial affinity to oxygen and is able to express under limited oxygen condition (Borisov et al., 2007). Furthermore, gene encoding for subunit H of the vacuolar H^+^-ATPase was absence in the microbial population of traditional Chinese soy sauce. Inspection of the glycolysis pathway revealed that the presence of gene encoding for the production of L-lactate (EC 1.1.27) and D-lactate (EC 1.1.1.28) dehydrogenase displayed fivefold decrease from day 0 to the sixth month of the fermentation process, correlating strongly with the relative abundance of LAB (Pearson’s correlation coefficient = 0.99; *p* < 0.00001). The abundance of genes coding for branched-chain amino acid aminotransferase (EC 2.1.6.42) in valine, leucine, and isoflavones biosynthesis and degradation increased gradually after the first month (**Figure 5**) with a strong positive correlation with the relative abundance of yeast (Pearson’s correlation coefficient = 0.81; *p* = 0.028). Although the ethanol concentration throughout the fermentation presented a moderate correlation with respect to the relative abundance of LAB (Pearson’s correlation coefficient = -0.69) and yeast (Pearson’s correlation coefficient = 0.68), the *p*-value was not low enough to be statistically significant.

## DISCUSSION

Whole genome shotgun is a valuable molecular tool to study the microbial dynamics and functionality in a given natural environmental habitat without the limitation and biases of culturing (Dunbar et al., 1997; Pettengill et al., 2012). In this study, WGS metagenomic via NGS approach was used to establish the natural microbiota succession and their functional capabilities involved in the traditional Chinese soy sauce fermentation brine in addition to determining its relation to physicochemical changes.

Our NGS data revealed a succession of microbial assemblage in the traditional Chinese soy sauce fermentation brine. In the first half of the soy sauce fermentation, bacteria were the dominant microorganism but succeeded subsequently by yeast. Notably, albeit a minority, food-borne pathogens from the genus *Salmonella* and *Listeria* (Farber and Peterkin, 1991; Mead et al., 1999) were present in the soy sauce mash. As these bacteria represent only a small portion of the microbial population in the soy sauce fermentation brine, it was considered as contaminant from the environment, most probably by the materials handlers. Their inability to survive in the fermentation tank was probably due to the stressful environment during the fermentation process and the production of antimicrobial peptides and proteins by the LAB (Paul Ross et al., 2002; Rattanachaikunsopon and Phumkhachorn, 2010).

The gradual decrease of pH values and steady increase of acidity content over the 6-month period of soy sauce fermentation, correlated to the presence of LAB. Our finding on the abundance of LAB present in our samples is in agreement with a previous study that has reported the isolation of LAB from soy sauce mash (Tanasupawat et al., 2002). It has been well documented- that these salt tolerant LABs are responsible for breaking down carbohydrate into lactic acid and simple sugar, which caused acidification of the mash (Kandler, 1983; Leroy and De Vuyst, 2004; Zhao et al., 2009). The decrease of pH level in the fermentative brine produced a hostile environment for less acid tolerant microorganism. In addition to LAB, yeasts, *Micrococcus*, *Streptococcus*, *Bacillus,* and related bacteria were present in the fermentation brine. Similar results were also reported previously to be present in soy sauce mash (Yokotsuka and Sasaki, 1997; Fukushima, 2004). However, it is noteworthy to point out that these published works are based on culturable bacteria study instead of using NGS as in the present work, which covers the unculturable microorganism.

The increase in the brine salinity and acidity as the fermentation progressed provided an optimal growth environment for halotolerant and acid tolerant yeast (Betts et al., 1999; van der Sluis et al., 2001). The present study observed an increase of the yeast population by more than 70% in which primarily involved the genera *Candida*, *Starmerella,* and *Wickerhamiella*, whilst there was a reduction of LAB abundance by 50% by the end of the fermentation process. Although the yeast population increased, the overall richness of the fungal population decreased over time. Interestingly, although the abundance of bacteria declined as the fermentation process proceeded, the richness of the bacterial population increased in the final stage of fermentation. This is due to the emergence of acid tolerant bacteria from the family Propionibacteriaceae (Jan et al., 2000) and Acidobacteriaceae (Hamberger et al., 2008). This correlates with the high acidic environment of the soy sauce fermentation brine by the end of the process. This study reports for the first time the presence of *Propionibacteriaceae* and *Acidobacteriaceae* in Chinese soy sauce fermentation. However, their existence only in the final month of fermentation may proof that they do not play an active role in the fermentation process. Further investigations are required to determine this. It is worthy to note that *Propionibacterium* has been used in the dairy industry as a secondary microflora to produce aromatic compounds and carbon dioxide (Giraffa, 2004).

By the end of the fermentation process, we observe the presence of yeast genus *Saturnispora*. This yeast was reported to have been isolated from a broad array of environment. This includes estuarine water from mangrove forest, flowers, forest soil, insect frass, marsh water, rhizosphere of oyster grass, and even *Drosophila* flies. This yeast has never been associated with soy sauce fermentation before, although it has been isolated from Sauerkraut (Barriga, 2011). Its function in biotechnology is unknown and raises a possibility of being a potential spoilage microorganism (Kurtzman et al., 2011). Thus, our results suggest that extending fermentation time may encourage growth of spoilage microbial population.

The LAB found in the present study can be divided into two groups established according to the end product of glucose fermentation. *Lactococcus, Enterococcus,* and *Pediococcus* are homofermentatives that produce sole lactic acid as the final product through Embden-Meyerhof-Parnas (EMP) pathway (Rattanachaikunsopon and Phumkhachorn, 2010). This explains the presence of genes encoded for the production of phosphofructokinase, pyruvate kinase, and lactate dehydrogenase, which are crucial in the EMP pathway. *Weissella, Leuconostoc,* and some of the lactobacilli are obligate heterofermentative LAB, which produced lactate, carbon dioxide, and ethanol and/or acetate as a result of hexose monophosphate or pentose pathway (Von Wright and Axelsson, 2004; König and Fröhlich, 2009). Furthermore, the observed gene abundance for the production of both D-lactate and L-lactate dehydrogenases decreased as the fermentation process progressed. This exhibited the shift to heterolactic fermentation (Moat et al., 2003) in the fermentative brine, which confirms that traditional Chinese soy sauce in the present study is achieved mostly by heterofermentative LAB.

We observed increased presence of gene encoding for the production of glucoamylase (EC 3.2.1.3) in the first month. This corresponded with increased concentration of reducing sugar in the first month as the enzyme hydrolyzed dextrin into simple sugars (Nigam and Singh, 1995). Meanwhile, the decrease in reducing sugar content by the fourth month suggested that the rising yeast population utilized the simple sugars as carbon source. Similar findings have also been observed by Chou and Ling (1998).

The emergence of yeast group in our samples correlated to the detection of ethanol in the samples. The yeast population utilized simple sugars and produced ethanol as by-products. This finding confirms the observation of Ghose and Bandyopadhyay (1980). The absence of *Zygosaccharomyces* and *Saccharomyces*, which commonly attributed to the production of ethanol in fermented product (Hamada et al., 1990; Nissen et al., 2000), explains the lower amount of ethanol in Chinese soy sauce brine than that of the popular Japanese *Koikuchi shoyu* (Luh, 1995; O’toole, 1997).

The presence of genes encoding for the branched-chain amino acids (BCAAs) – isoleucine, leucine and valine were observed from the soy sauce mash. BCAAs were reported to be important in some LAB for proteolysis (Liu et al., 2008) and essential in the production of volatile compounds such as acids, alcohol and esters (Yvon and Rijnen, 2001; Marilley and Casey, 2004; Smit et al., 2005). *Candida* species are able to metabolize the BCAAs through the Ehrlich pathway, which involves the branched-chain aminotransferase, decarboxylase, and alcohol dehydrogenase in producing flavor compounds in fermented food products (Derrick and Large, 1993; ter Schure et al., 1998). The gene encoding for the production BCAA aminotransferase increased as the fermentation process progressed. This corresponds to the increased abundance of *Candida* in our sample, which was found to be the most abundant microorganism by the end of the fermentation process. Interestingly, *Candida* was reported as producing higher amount of flavoring substances as compared to other yeasts (O’toole, 1997).

The present study provides evidence of adaptive potential, especially in relation to oxidative phosphorylation. The presence of cytochrome *bd* complex coupled with high level of coverage in pentose phosphate pathway indicated reaction toward low oxygen level in the fermentation tank. Furthermore, there was an absence of gene encoding for subunit H of the vacuolar H^+^-ATPase. Subunit H is not required for the assembly of V-ATPase but an ATPase devoid of it cannot function and are unable to pump protons into the cell (Nishi and Forgac, 2002; Alba-Lois and Segal-Kischinevzky, 2010).

It was reported that lacking V-ATPase activity in yeast is unable to grow at pH neutral media, but thrive under a more acidic environment (Graham et al., 2003). This correlates back to our study, with the acidic condition in our soy sauce mash.

To the author’s knowledge, this is the first microbiota studied using NGS, reporting both the taxonomy and the microbial functional potential throughout traditional Chinese soy sauce fermentation process for a total of 6 months.

The small sample size in this study prevents us from concluding results representing the whole traditional Chinese soy sauce fermentation process. However, this study does allow an overview of the complexities involved in this “ancient biotechnology” fermentation practice. Our meta-data showed the pH was closely associated with the abundance of LAB detected. The production of ethanol corresponded well to the shift to heterotrophic fermentation and the depletion of reducing sugar, in the spontaneous presence of yeast population. In addition to that, the total nitrogen liberated from soy and wheat flour protein increased without the addition of exogenous supply of nitrogenous sources. Future metatranscriptomic studies are required to support our findings in regards to functional properties during the fermentation process. Though WGS sequencing is still too costly for standard use in the food industry, we do believe that the rapid lowering of sequencing costs and automation will result in it becoming a routine technology, employed for food quality, and insights into microbial activities in food samples.

## AUTHOR CONTRIBUTIONS

Joanita Sulaiman performed experiments, analyzed data, and wrote the paper; Wai-Fong Yin design experiments, prepared the samples, edited the paper; Han Ming Gan analyzed and interpret data; Kok-Gan Chan design, supervised the project, and wrote the paper.

## Conflict of Interest Statement

The authors declare that the research was conducted in the absence of any commercial or financial relationships that could be construed as a potential conflict of interest.
